# A Renewable Sedimentary Slurry Battery: Preliminary Study in Zinc Electrodes

**DOI:** 10.1016/j.isci.2020.101821

**Published:** 2020-11-19

**Authors:** Yue Liu, Qiyang Hu, Jing Zhong, Zhixing Wang, Huajun Guo, Guochun Yan, Xinhai Li, Wenjie Peng, Jiexi Wang

**Affiliations:** 1 School of Metallurgy and Environment, Central South University, Changsha, 410083, P.R. China; 2 Engineering Research Center of the Ministry of Education for Advanced Battery Materials, Central South University, Changsha 410083, PR China; 3 State Key Laboratory for Power Metallurgy, Central South University, Changsha 410083, PR China

**Keywords:** Energy Materials, Energy Storage, Energy Systems, Materials Characterization

## Abstract

Low-cost, scalable energy storage is the key to continuing growth of renewable energy technologies. Here a battery with sedimentary slurry electrode (SSE) is proposed. Through the conversion of discrete particles between sedimentary and suspending types, it not only inherits the advantages of semi-solid flow cell but also exhibits high energy density and stable conductive network. Given an example, the zinc SSE (ZSSE) delivers a large discharge capacity of 479.2 mAh g^−1^ at 10 mA cm^−2^. More importantly, by renewal of the slurry per 20 cycles, it can run for 112 and 75 cycles before falling below 80% of designed capacity under 10 mA cm^−2^ (20% DOD_Zn_) and 25 mA cm^−2^ (25% DOD_Zn_), respectively. The lost capacity after cycles is able to recover after slurry renewal and the end-of-life SSE can be easily reused by re-formation. The concept of SSE brands a new way for electrochemical energy storage.

## Highlights

• A renewable semi-solid sedimentary slurry battery is proposed
• Lost capacity after cycles is able to recover after slurry renewal
• End-of-life SSE can be easily reused by re-formation

## Graphical Abstract

**Figure:**
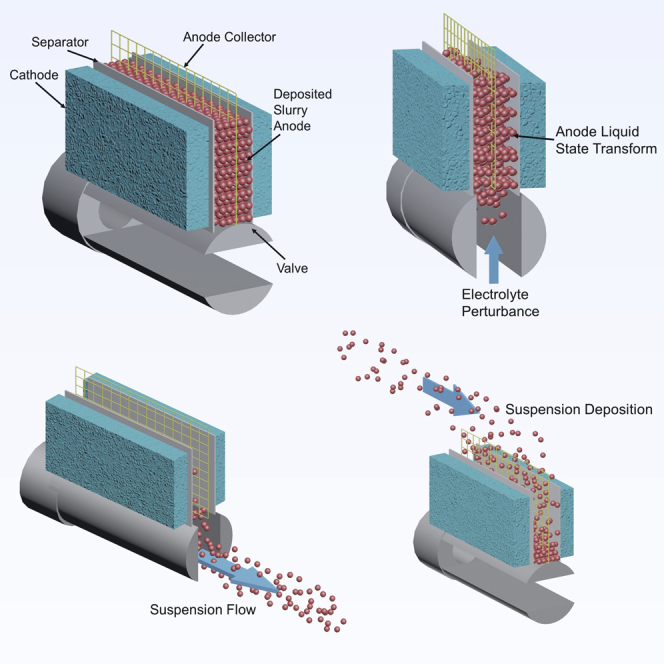

## Introduction

Sustainable energy sources are urgently needed owing to the environmental pollution caused by fossil fuel consumption and ecological damage (Dunn et al., 2011; Leng et al., 2019). Nowadays, low-cost, scalable energy storage is the key to continuing growth of renewable energy technologies (Ferrara et al., 2019; Kamat et al., 2017; Lin et al., 2015). Benefitting from the remarkable merits of flowability of active material, redox flow batteries realize rapid charging by updating the electrolyte and decoupling of power unit and energy unit (Ding et al., 2018; Li et al., 2015a; Luo et al., 2019; Pan and Wang 2015; Wang et al., 2013; Zhou et al., 2019). Hence, it has been widely used in the large-scale stationary energy storage applications (Ke et al., 2018; Wei et al., 2016; Yuan et al., 2019). However, limited by the low ion solubility, conventional redox flow batteries show poor energy density (Duan et al., 2017; Duduta et al., 2011; Yang et al., 2018). If the energy density can be greatly increased, the ideal redox flow batteries enable unprecedented models for electrical storage, which may revolutionize the existing pattern of energy utilization. For instance, one can realize rapid refueling of vehicles by replacing the flowable active substance of batteries (Chen et al., 2017; Wang et al., 2012). It is an effective way to increase the energy density of the redox flow battery through turning the active material dissolved in the electrolyte into a solid particulate (Chen and Lu 2016; Li et al., 2015b). Using this approach, Y.M. Chiang's group demonstrated a new type of flow battery technology called semi-solid flow cell (SSFC) (Duduta et al., 2011). The semi-solid electrode is a stable suspension that is made by mixing the active material, carbon black, and electrolyte. Then the suspension is injected into the reaction chamber for the charge and discharge reaction. In this way, the energy density of SSFC reaches the level of the lithium-ion battery. Not only in lithium-ion battery material, the conception of SSFC can even apply in vanadium redox flow batteries (Lohaus et al., 2019; Percin et al., 2018) and zinc-nickel batteries (Liu and Wang 2015). Unfortunately, the energy density of SSFC is contradictory to its slurry fluidity. When the active substance content is increased in the suspension, the energy density of the battery will be enlarged while the fluidity of the slurry will deteriorate seriously. In addition, the conductive network of SSFC is composed by the dynamic carbon black chain that is so-called percolating network (Duduta et al., 2011; Rommerskirchen et al., 2019), and its electronic conductivity is unstable. As a result, the cell exhibits poor cycle performance at large current density (Chen et al. 2015, 2017). Therefore, the SSFC needs a proper solid content to reach a balance between high energy density, acceptable conductivity, and low viscosity (Akuzum et al., 2020).

Sedimentation and movement of particulate materials are fairly common phenomena in daily life and industrial production, such as slurry transportation, hourglass, soil liquefaction, quicksand, and alluvial plain formation. Particulate cluster composed by numerous particles is a critical phase between solid and liquid. Under the effect of gravity, it generally maintains a solid state with a stable sedimentation structure. However, the structural relaxation and collapse occur when the sedimentation structure is subjected to a small external perturbation. As a result, the particulate cluster behaves like liquid (Kou et al., 2017).

**Figure 1:**
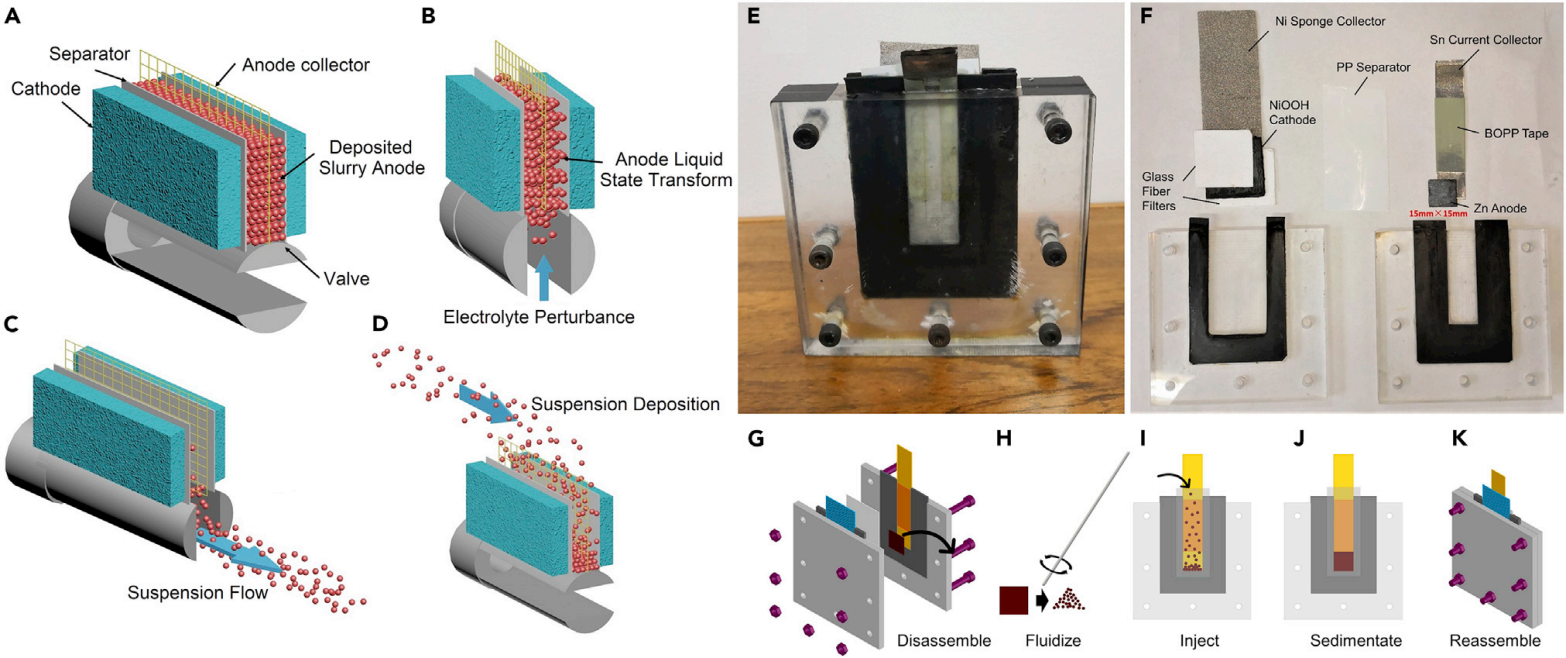
Sedimentary Slurry Electrodes (SSE) and Simulated Slurry-Renewal Process (A–D) Schematic diagram of the sedimentary slurry electrodes (SSE); (E, F) photos of (E) preliminary mold for the zinc sedimentary slurry electrode (ZSSE) mold and (F) corresponding internal structure; (G–K) flow chart of the simulated slurry-renewal process experiencing disassembling, fluidizing, injecting, sedimentating and reassembling.

Based on the solid-liquid two-phase transformation ability of the particulate cluster and the transportability of the particulate suspension, we propose an electrode structure with the characteristics of a semi-solid flow battery, called as the sedimentary slurry electrode (SSE). The slurry is a mixture of active material particles and electrolyte. Herein, two forms of slurry are included: suspension slurry and sedimentary slurry. A working state electrode is formed by stacking of discrete particles named sedimentary slurry (Figure 1A). The stacking particulate group will liquefy and gain fluidity after mixing with extra electrolyte (Figure 1B). When the slurry moves rapidly, the solid particulate in the slurry suspends in the electrolyte to form the stable suspension, realizing the speedy transfer of the active material (Figure 1C). We call this state as suspension slurry. The sedimentation process of the particles occurs under the gravity effect when the flow rate of the slurry slows down or stops consequently (Figure 1D). As a result, the sedimentary slurry is formed again. Through this process, theoretically, SSE has a high energy density and a stable conductive network in the sedimentary slurry state. Moreover, the conversion of the sedimentary slurry to the suspension slurry can achieve an excellent fluidity with less energy loss of the system.

## Results

### Simulation of Slurry-Renewal Process

To characterize the electrochemical performance of proposed SSE in primary and secondary batteries, a preliminary experimental mold (Figures 1E, 1F) is demonstrated with zinc sedimentary slurry electrode (ZSSE) and commercial NiOOH as anode and cathode, respectively. This particulate sedimentary structure electrode is expected to exhibit excellent electrochemical performance. Particularly, the lost capacity of the battery is designed to be recovered by renewing the slurry. With such unique structure, ZSSE may effectively solve the dendritic problem of zinc batteries at high rate and prolong the life of metal electrode. Furthermore, a simple process to simulate the slurry renewal process of SSE is designed for simulating the conductive network reconstruction in SSE concept (Figures 1G–1K). At the beginning of renewal, the sedimentary slurry is taken out after disassembling the mold (Figure 1G). Then the slurry is stirred in the beaker, turning it into the suspension slurry (Figures 1H, 1I). After that, the obtained suspension slurry is injected into the negative chamber and sedimentated to form the sedimentary slurry (Figure 1J). Finally, the battery mold is reconstructed for electrochemical tests (Figure 1K). Such whole renewal process is called as first renewal. Before formal cycling experiment, the cells were stood for 30 min to ensure that the slurry settles completely. When the cycle is over, the second renewal begins, and so on.

### Formation Process of ZSSE

**Figure 2:**
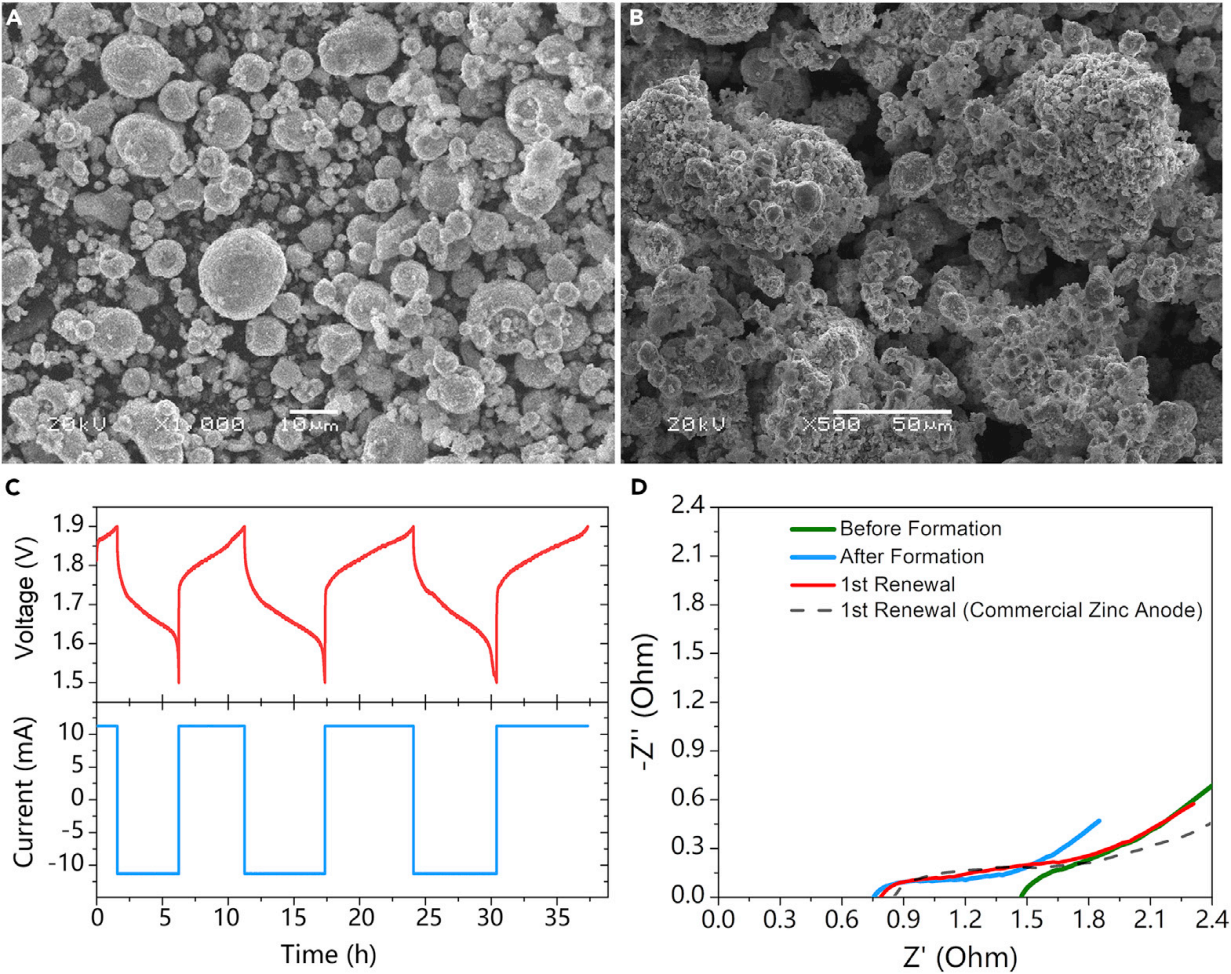
Characterization for Formation of ZSSE (A, B) SEM images of slurry particulate (A) before formation process and (B) after three rounds of formation; (C) voltage versus current curves of the battery formation process at 5 mA cm^−2^ in the voltage range of 1.5–1.9 V; (D) EIS before and after formation and after first renewal.

Before the formation process, the powders are shaped as spheres (Figure 2A). After the formation process, the powder particulates agglomerate into large particles. At the same time, the formation process converts the particle surface from smooth to rough (Figure 2B). As a result, the specific surface area of the electrode increases from 1.08 to 6.20 m^2^ g^−1^, which is beneficial to enhancing the electrical conducting between the particles and thus improving the reversibility of the zinc anode. Furthermore, the particulate packing structure has abundant packing voids, promoting the ion transfer process inside the ZSSE. In addition, the packing voids are filled with electrolyte, keeping the particulate cluster in a non-equilibrium state. As a result, the SSE can be easily liquefied with slight stir in the renewal process. As shown in Figure 2C, the capacity is continuously increased during the formation process, indicating that more active materials can be utilized. In addition, the ohmic resistance after formation decreases significantly from ~1.5 to ~0.8 ohm (Figure 2D), verifying the increased conductivity of the zinc electrode after formation. Note that there is no obvious change in ohmic resistance between ZSSE and commercial zinc anode. That is to say, with good conductivity of the metal, the point-to-point contact does not significantly affect the conductivity of the electrode.

### Electrochemical Characterization and Slurry Renewal of ZSSE

**Figure 3:**
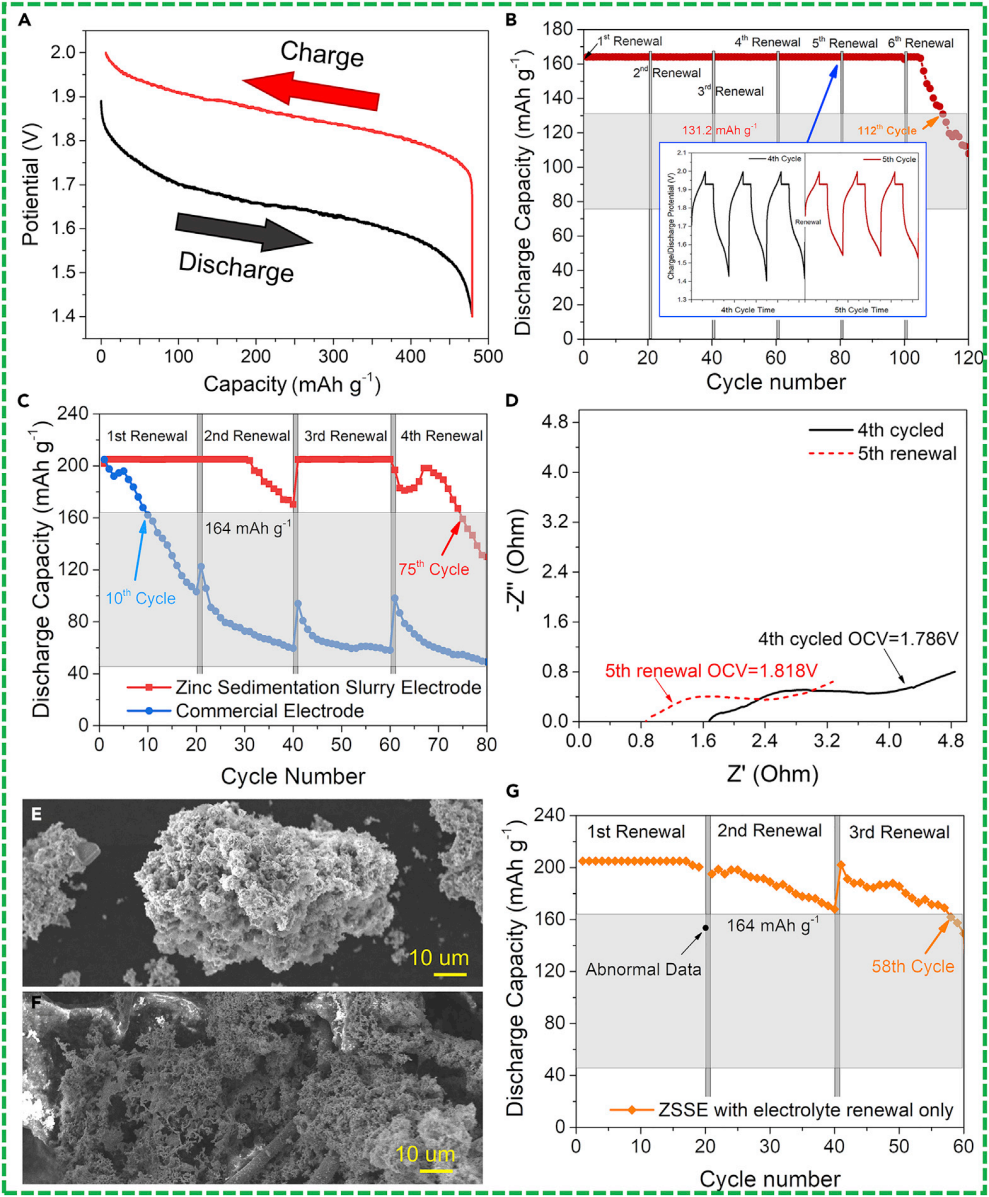
Electrochemical Performance and Morphological Change of ZSSE (A) Charge-discharge curve of ZSSE; (B) discharge capacity curves upon limited capacity cycles (20% DOD_Zn_, 10 mA cm^−2^) of ZSSE; (C) discharge capacity curves upon limited capacity cycles (25% DOD_Zn_, 25 mA cm^−2^) of ZSSE and commercial zinc anode; (D) EIS of ZSSE at different periods during cycling; (E, F) SEM images of (E) ZSSE and (F) commercial zinc electrode after 80 cycles of limited capacity (25% DOD_Zn_, 10 mA cm^−2^) tests; (G) discharge capacity curves upon limited capacity cycles (25% DOD_Zn_, 10 mA cm^−2^) of ZSSE with renewal of electrolyte only.

Matching with the air cathode in a primary battery is one of the applications of ZSSE; the metal slurry can be injected into the battery as a "fuel" to realize mechanical energy charging. Although several similar studies in zinc fuel cells field have been reported (Pei et al., 2014; Sapkota and Kim 2009), it has to be admitted that in our concept of ZSSE, the active material is recyclable, which is distinctly different from that being completely consumed in the reaction chamber. As such, the ZSSE can make the active material in different regions consistent basically by the slurry renewal, which is fundamental to the stability of the system. The ability of ZSSE to discharge to high Zn mass-normalized capacity and be recharged without inducing dendritic shorts is probed by exhaustively discharging the cells with ZSSE at a current density of 10 mA cm^−2^ and then recharging at the same rate. A typical discharge voltage platform between 1.7 and 1.5 V is presented in Figure 3A, which shows a stable discharge voltage without sudden drop, indicating that the conductive network of ZSSE is relatively stable during discharge. The discharge capacity of the test battery reaches around 479.2 mAh g^−1^ (58.44% DOD_Zn_) and is able to recharge to >98% of capacity from these extreme depths.

Zinc-nickel secondary battery suffers from the passivation and uneven deposition of the zinc during the cycle. Since SSE has the ability to reconstruct the conductive network, the spatial position of the active particulate is not fixed during the working process, so that the problem of uneven deposition of the zinc negative electrode can be solved theoretically. Generally, the normal discharge depth of the secondary zinc anode is about 20% of the theoretical zinc anode (20% DOD_Zn_). Therefore, the discharge capacity of the cycle tests is limited to 20% DOD_Zn_ (164 mAh g^−1^) to probe the cycle performance of the ZSSE under a modest current density and a normal discharge depth. To ensure that the current collector would not be decomposed, 2.0 and 1.4 V are set as the charge and discharge cut-off voltage, respectively. As such, when the battery cannot cycle at the specified capacity, the battery capacity will be reduced owing to the cut-off voltage we set. The current density is 10 mA cm^−2^ in both galvanostatic charge or discharge processes. In addition, in order to ensure that the NiOOH electrode is completely oxidized but O_2_ evolution is avoided, the constant potential charging process should be performed at 1.93 V at the end of constant-current charging process (Bonnick and Dahn 2012). Slurries are updated in every 20 cycles. As shown in Figure 3B, the cell runs for 105 cycles at the modest current density and cycle capacity condition, almost without the capacity lost. Its life ends at 113 cycles when the capacity attenuates to 80% of its original one. In addition, it can be seen from the charge-discharge voltage curves (inside of Figure 3B) that the discharge termination voltage at the end of discharge shows a significant improvement after slurry renewal, reflecting that the slurry-renewal process in SSE conception makes the lost overpotential recovered.

To investigate the cycle ability of the ZSSE at larger current density and deeper discharge depth, the following experiment increases the discharge current density to 25 mA cm^−2^ and limits the discharge capacity at 25% DOD_Zn_ (205 mAh g^−1^). A control group using a commercial zinc anode, which is a porous electrode with active materials tightly bound together, is set to observe the surface dendrite situation. The only difference between the experiments of ZSSE and commercial zinc anode is the renewal process. The commercial zinc anode will be taken out and soaked in the electrolyte for 5 min to realize the electrolyte renewal just as the slurry-renewal process does. As shown in Figure 3C, the commercial zinc anode cannot work stably in this condition. The capacity declines rapidly and stabilizes at around 60 mAh g^−1^. If the battery life is defined as the number of cycles when the battery capacity decays to 80% of the original capacity, the life of a commercial zinc anode is only 10 cycles. As a comparison, the life of the ZSSE under this condition reaches 75 cycles. Moreover, Figure 3C also indicates that both types of electrodes get attenuation in this cycle condition and the electrolyte updating and slurry renewal can reduce the capacity attenuation. However, the capacity recovery of the ZSSE is significantly better than that of the commercial zinc anode, which can greatly prolong the life of the ZSSE. This can be further confirmed by EIS and SEM results (Figures 3D–3F). As shown in Figure 3D, the internal resistance of battery increases with the battery capacity decay and the renewal step can significantly reduce the ohmic impedance of the electrode to the original level. SEM images of the cycled ZSSE and the cycled commercial zinc anode are shown in Figures 3E and 3F, respectively. After cycling, the surface of the zinc particles of ZSSE changes slightly. The granular morphology remains, and no dendrite can be found on the surface. In addition, the macroporous structure of the particles is cleared presented. However, the commercial zinc anode exhibits dense zinc whiskers, and the original pores on the surface are blocked owing to uneven deposition of zinc, resulting in irreversible attenuation of capacity and even short circuit.

In order to investigate the actual impact of slurry renewal and electrolyte renewal on electrode capacity recovery, an experiment is designed that the ZSSE is slowly rinsed by the fresh electrolyte, instead of going on with the slurry-renewal process when 20 cycles are finished. This process neither takes out the active material nor reconstructs the electrode conductive network. The experimental conditions in Figure 3G are completely consistent with that in Figure 3C (25% DOD_Zn_, 25 mA cm^−2^). Figure 3G shows the discharge special capacity of the ZSSE with electrolyte renewal only. The above-mentioned phenomenon like resistance reducing and loss capacity recovering happens again, indicating that the composition of the electrolyte can greatly affect the cycle stability of the battery. This result is consistent with the conclusions in the experiments related to the single flow zinc/nickel battery (Zhang et al., 2008). However, comparing with the electrolyte renewal simply, the overall renewal of the slurry has a higher capacity recovery ability, which indicates that the reconstruction of the electrode conductive network has a unique effect on the electrode capacity recovery.

## Discussion

### Working Mechanism of ZSSE

**Figure 4:**
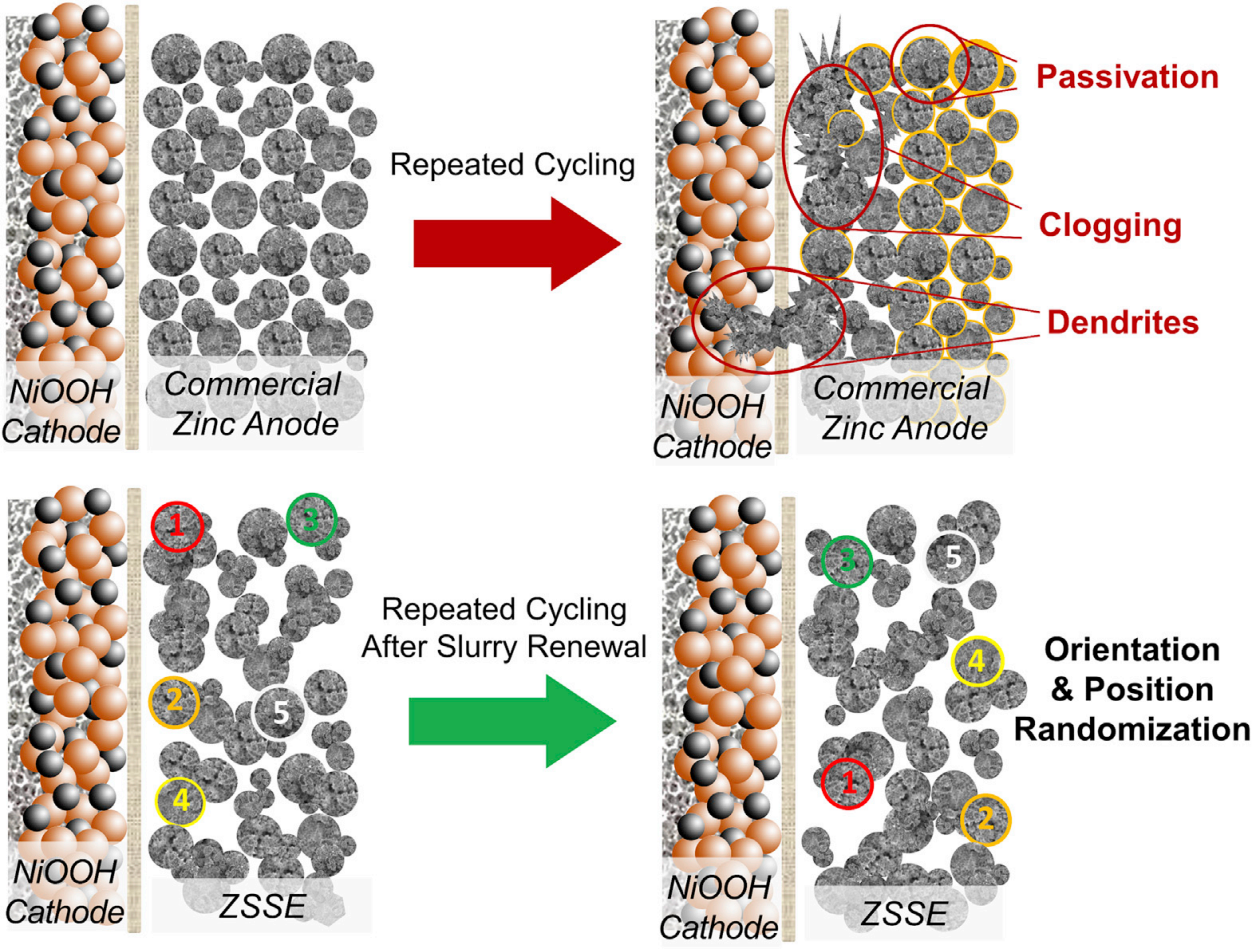
Working Mechanism Differences between Commercial Zinc Anode and ZSSE The slurry-renewal process for ZSSE reconstructs the conductive network and randomizes the spatial position of the active material particles, whereas the commercial zinc anode only replaces the electrolyte on the electrode surface by electrolyte refreshing.

Figure 4 shows the working mechanism differences between commercial zinc anode and ZSSE. It explains that the electrolyte deeply inside the electrode deteriorates faster than that at the surface owing to the ion migration distance, and a large amount of active materials cannot fully participate in the electrochemical reaction. The slurry-renewal process reconstructs the conductive network and randomizes the spatial position of the active material particles, which refreshes the electrolyte environment around the zinc particulate inside the electrode and exposes the interface of the active material that does not react completely inside the electrode. As such, the ZSSE has less area where the electrolyte is liable to deteriorate in the electrode comparing with the commercial zinc anode, and thus the active material in the electrode can be better utilized. However, the commercial zinc electrode only replaces the electrolyte on the electrode surface by electrolyte refreshing. As a result, ZSSE delivers higher capacity durability than the commercial zinc anode.

### Deep Utilization, Frequent Slurry Renewal, and Simple Reuse of ZSSE

**Figure 5:**
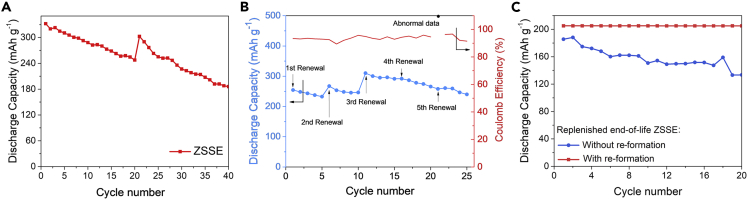
Discharge Capacity Curves of ZSSE (A) Discharge capacity curves of ZSSE without limited capacity (2.0-1.4 V, 25 mA cm^−2^); (B) discharge capacity curve of ZSSE under increasing renewal frequency condition; (C) discharge capacity curves of the ZSSE whose slurry is the life-of-end slurry but that underwent re-formation, compared with the ZSSE replenished end-of-life slurry without re-formation.

For zinc-nickel secondary battery which used aqueous electrolyte, deep charge/discharge in large current density is obviously unfavorable for battery performance, which will lead to rapid decline for the battery capacity. A harsh condition is chosen to detect the characteristics of ZSSE. The galvanostatic charge and discharge current densities are 10 and 25 mA cm^−2^, respectively. The renewal frequency is consistent with previous experiments (once every 20 cycles), but the battery is discharged as deep as possible without capacity limit. As shown in the Figure 5A, the initial capacity of ZSSE reaches 332.2 mAh g^−1^, and its capacity declines rapidly at this condition, the capacity remains 74.6% after 20 cycles. Slurry-renewal process make the declined capacity restore, returning to 91.1% of initial capacity.

Previous experiments infer that the slurry-renewal process refreshes the electrolyte environment of the electrode, so that the capacity of the electrode can recover to a certain extent. It is reasonable to speculated that the capacity of ZSSE can be maintained at a high level theoretically if the frequency of slurry-renewal increases. In order to investigate the effect of increasing the frequency of slurry renewal on the capacity retention of the battery, ZSSE is tested under the conditions of increasing the renewal frequency, enlarging the current density and cycling deeply. The charge/discharge currents and the cut-off window are identical with the previous experiments (10 mA cm^−2^, 25 mA cm^−2^, 2.0-1.4 V), but slurry renews every 5 cycles. As shown in Figure 5B, the battery undergoes 5 times of slurry-renewals for a total of 25 charge and discharge cycles. The capacity always maintains above 232 mAh g^−1^, revealing that the method can be practicable comparing with that in Figure 5A. The discharge capacity increase caused by the slurry renewal is obvious, it is normal that the capacity of the slurry after the renewal is higher than the initial capacity of the front cycle, because the capacity increase represents an increase in the utilization of active substances, and it hardly maintains consistently under the condition of limited voltage window. The capacity of the electrode cannot be completely recovered during the last few slurry-renewal processes. It may be on account of that the process of slurry-renewal would take out the tiny zinc particulate inevitably, probably causing sedimentary slurry loss and thus weakening the ability of the capacity recovery. Actually, the material loss is more than 40% after 4 times slurry-renewal process. However, the material loss is not an inherent issue of the SSE conception. When the device is integrated, instead of using a variety of independent equipment to simulate the slurry-renewal process as in this experiment, such problem can be avoided.

Traditionally, end-of-life batteries need to be disassembled and the raw materials are recovered by metallurgical methods. In the SSE concept, the sedimentary slurry in the battery can be flowed by transforming it into the suspension slurry. It means that the end-of-life slurry can be taken out of the battery easily. If the reuse of waste slurry can be achieved directly instead of being used as waste to produce raw metal materials, the cost of SSE can be further reduced. This idea is verified through experiments. Specially, the slurry is taken out from several batteries that have been run for more than 100 cycles, and reloaded into one cell to ensure that the slurry quality reaches the original level. The capacity limit test condition is same as before. As shown in Figure 5C, the battery is not able to restore capacity as much as the original one (blue line), indicating that after repeated slurry renewal processes, not only the mass of the active material gets lost, but also the capacity of the slurry itself has attenuation after cycling. When the slurry is taken out, it is soaked and washed with deionized water, dried in a vacuum oven at 80°C for 4h, grinded as fine as possible by a mortar. Then, the formation process runs again, and the reprocessed slurry shows almost the same performance as the new slurry (compared with Figure 3C). It leads to a promising possibility, that is, when the SSE scales up to the integrated level, the end-of-life slurry may not need to be re-produced through a complicated process. Instead, the reuse of materials can be realized just through simple electrochemical re-formation treatment of the end-of-life slurry.

### Limitations of the Study

Because of the rough mold and low automation at lab, the single experiment takes a long time and the experiment error is relatively large. In addition, the manual simulation of renewal process would inevitably lead to the loss of active materials, which affects the reliability of the experimental conclusion at the end of pulp life.

### Resource Availability

#### Lead Contact

Further information and requests for resources and reagents should be directed to and will be fulfilled by the Lead Contact, Jiexi Wang (wangjiexikeen@csu.edu.cn).

#### Materials Availability

This study did not generate new unique reagents.

#### Data and Code Availability

This study did not generate datasets or analyze codes.

## Methods

All methods can be found in the accompanying Transparent Methods supplemental file.

## Supplemental Information

Document S1. Transparent Methods

## References

[bib1] Akuzum B., Singh P., Eichfeld D.A., Agartan L., Uzun S., Gogotsi Y., Kumbur E.C. (2020). Percolation characteristics of conductive additives for capacitive flowable (semi-solid) electrodes. ACS Appl. Mater. Interfaces.

[bib2] Bonnick P., Dahn J.R. (2012). A simple coin cell design for testing rechargeable zinc-air or alkaline battery systems. J. Electrochem. Soc..

[bib3] Chen H., Lai N.-C., Lu Y.-C. (2017). Silicon–carbon nanocomposite semi-solid negolyte and its application in redox flow batteries. Chem. Mater..

[bib4] Chen H., Lu Y.-C. (2016). A high-energy-density multiple redox semi-solid-liquid flow battery. Adv. Energy Mater..

[bib5] Chen H., Zou Q., Liang Z., Liu H., Li Q., Lu Y.C. (2015). Sulphur-impregnated flow cathode to enable high-energy-density lithium flow batteries. Nat. Commun..

[bib6] Ding Y., Zhang C., Zhang L., Wei H., Li Y., Yu G. (2018). Insights into hydrotropic solubilization for hybrid ion redox flow batteries. ACS Energy Lett..

[bib7] Duan W., Huang J., Kowalski J.A., Shkrob I.A., Vijayakumar M., Walter E., Pan B., Yang Z., Milshtein J.D., Li B. (2017). “Wine-dark sea” in an organic flow battery: storing negative charge in 2,1,3-benzothiadiazole radicals leads to improved cyclability. ACS Energy Lett..

[bib8] Duduta M., Ho B., Wood V.C., Limthongkul P., Brunini V.E., Carter W.C., Chiang Y.-M. (2011). Semi-solid lithium rechargeable flow battery. Adv. Energy Mater..

[bib9] Dunn B., Kamath H., Tarascon J.M. (2011). Electrical energy storage for the grid: a battery of choices. Science.

[bib10] Ferrara M., Chiang Y.-M., Deutch J.M. (2019). Demonstrating near-carbon-free electricity generation from renewables and storage. Joule.

[bib11] Kamat P.V., Schanze K.S., Buriak J.M. (2017). Redox flow batteries. ACS Energy Lett..

[bib12] Ke X., Prahl J.M., Alexander J.I.D., Wainright J.S., Zawodzinski T.A., Savinell R.F. (2018). Rechargeable redox flow batteries: flow fields, stacks and design considerations. Chem. Soc. Rev..

[bib13] Kou B., Cao Y., Li J., Xia C., Li Z., Dong H., Zhang A., Zhang J., Kob W., Wang Y. (2017). Granular materials flow like complex fluids. Nature.

[bib14] Leng J., Wang Z., Wang J., Wu H.H., Yan G., Li X., Guo H., Liu Y., Zhang Q., Guo Z. (2019). Advances in nanostructures fabricated via spray pyrolysis and their applications in energy storage and conversion. Chem. Soc. Rev..

[bib15] Li B., Nie Z., Vijayakumar M., Li G., Liu J., Sprenkle V., Wang W. (2015). Ambipolar zinc-polyiodide electrolyte for a high-energy density aqueous redox flow battery. Nat. Commun..

[bib16] Li J., Yang L., Yang S., Lee J.Y. (2015). The application of redox targeting principles to the design of rechargeable Li–S flow batteries. Adv. Energy Mater..

[bib17] Lin K., Chen Q., Gerhardt M.R., Tong L., Kim S.B., Eisenach L., Valle A.W., Hardee D., Gordon R.G., Aziz M.J. (2015). Alkaline quinone flow battery. Science.

[bib18] Liu J., Wang Y. (2015). Preliminary study of high energy density Zn/Ni flow batteries. J. Power Sources.

[bib19] Lohaus J., Rall D., Kruse M., Steinberger V., Wessling M. (2019). On charge percolation in slurry electrodes used in vanadium redox flow batteries. Electrochem. Commun..

[bib20] Luo J., Hu B., Hu M., Zhao Y., Liu T.L. (2019). Status and prospects of organic redox flow batteries toward sustainable energy storage. ACS Energy Lett..

[bib21] Pan F., Wang Q. (2015). Redox species of redox flow batteries: a review. Molecules.

[bib22] Pei P., Ma Z., Wang K., Wang X., Song M., Xu H. (2014). High performance zinc air fuel cell stack. J. Power Sources.

[bib23] Percin K., Rommerskirchen A., Sengpiel R., Gendel Y., Wessling M. (2018). 3D-printed conductive static mixers enable all-vanadium redox flow battery using slurry electrodes. J. Power Sources.

[bib24] Rommerskirchen A., Kalde A., Linnartz C.J., Bongers L., Linz G., Wessling M. (2019). Unraveling charge transport in carbon flow-electrodes: performance prediction for desalination applications. Carbon.

[bib25] Sapkota P., Kim H. (2009). Zinc–air fuel cell, a potential candidate for alternative energy. J. Ind. Eng. Chem..

[bib26] Wang W., Luo Q., Li B., Wei X., Li L., Yang Z. (2013). Recent progress in redox flow battery research and development. Adv. Funct. Mater..

[bib27] Wang Y., He P., Zhou H. (2012). Li-redox flow batteries based on hybrid electrolytes: at the cross road between Li-ion and redox flow batteries. Adv. Energy Mater..

[bib28] Wei X., Duan W., Huang J., Zhang L., Li B., Reed D., Xu W., Sprenkle V., Wang W. (2016). A high-current, stable nonaqueous organic redox flow battery. ACS Energy Lett..

[bib29] Yang F., Mousavie S.M.A., Oh T.K., Yang T., Lu Y., Farley C., Bodnar R.J., Niu L., Qiao R., Li Z. (2018). Sodium–sulfur flow battery for low-cost electrical storage. Adv. Energy Mater..

[bib30] Yuan Z., Yin Y., Xie C., Zhang H., Yao Y., Li X. (2019). Advanced materials for zinc-based flow battery: development and challenge. Adv. Mater..

[bib31] Zhang L., Cheng J., Yang Y.-s., Wen Y.-h., Wang X.-d., Cao G.-p. (2008). Study of zinc electrodes for single flow zinc/nickel battery application. J. Power Sources.

[bib32] Zhou M., Chen Y., Zhang Q., Xi S., Yu J., Du Y., Hu Y.-S., Wang Q. (2019). Na_3_V_2_(PO_4_)_3_ as the sole solid energy storage material for redox flow sodium-ion battery. Adv. Energy Mater..

