# Supplementary material for: A Renewable Sedimentary Slurry Battery: Preliminary Study in Zinc Electrodes
Source: iScience. 2020 Nov 19;23(12):101821. doi: 10.1016/j.isci.2020.101821 (PMC7710632; doi:10.1016/j.isci.2020.101821)
Supplement: Document S1. Transparent Methods [file mmc1.pdf]

iScience, Volume *23*

Supplemental Information

A Renewable Sedimentary Slurry Battery: Preliminary Study in Zinc Electrodes

Yue Liu, Qiyang Hu, Jing Zhong, Zhixing Wang, Huajun Guo, Guochun Yan, Xinhai Li, Wenjie Peng, and Jiexi Wang

Supplemental File

A Renewable Sedimentary Slurry Battery: Preliminary Study in Zinc Electrodes

Yue Liu,[1] Qiyang Hu,[1,2] Jing Zhong,[1] Zhixing Wang,[1,2] Huajun Guo,[1,2] Guochun Yan,[1,2] Xinhai Li,[1,2] Wenjie Peng,[1,2] Jiexi Wang[1,2,3,]*

[a] School of Metallurgy and Environment, Central South University, Changsha, 410083, P.R. China.

[b] Engineering Research Center of the Ministry of Education for Advanced Battery Materials, Central South University, Changsha 410083, PR China

[c] State Key Laboratory for Power Metallurgy, Central South University, Changsha 410083, PR China

* Correspondence, E-mail: wangjiexikeen@csu.edu.cn

Transparent Methods

Materials preparation: Porous zinc particles used as the negative electrode in this work were prepared by an electrochemical approach called the formation step. Before the formation step, 11 wt.% of $Ca(OH)_2$ was added into the zinc powder (0.275g, diameter < 18 um) to reduce the solubility of Zn^{2+} and accelerate the dehydration/precipitation process (Parker et al. 2016, Yu et al. 2001). Then the constructed battery was cycled at a small current density (5 mA cm^{-2}) for 3 cycles deeply. After dried and grinded, the porous zinc particles with rich macroporous structure were obtained.

Nickel-zinc battery discharges accompanying with the oxidation of Zn anode and the reduction of NiOOH cathode, according to the anodic (Eqs. 1-3) and cathodic (Eq. 4) reactions. The electrolyte is a mixed solution of KOH (6 mol L^{-1}) and LiOH (1 mol L^{-1}) (Parker et al. 2017). The role of Li^+ is to suppress the evolution of O_2 on the positive electrode in order to improve the cathodic cycle performance.(Rubin and Baboian 1971) Excess ZnO powder were added into the electrolyte until it was saturated during the preparation, saturating $Zn(OH)_4^{2-}$ in the electrolyte could reduce the dissolution of the zinc anode in cycling.

Electrooxidation: $Zn(s) \rightarrow Zn^{2+}(aq) + 2e^-$ (1)

Complexation: $Zn^{2+}(aq) + 4OH^-(aq) \rightarrow Zn(OH)_4^{2-}(aq)$ (2)

Dehydration/Precipitation: $Zn(OH)_4^{2-}(aq) \rightarrow ZnO(s) + H_2O(l) + 2OH^-(aq)$ (3)

Electroreduction: $\beta\text{-}NiOOH(s) + 2H_2O(l) + 2e^- \rightarrow 2\beta\text{-}Ni(OH)_2(s) + 2OH^-(aq)$ (4)

The positive electrode was gained from the disassembled commercial zinc-nickel battery. It is a sintered NiOOH electrode which has been widely used. To match the negative electrodes capacity, the experimental positive electrode was produced by bonding two commercial cathodes and one piece of nickel foam under a pressure of 20 MPa followed by drying up. The active material was composed of PVP, conductive carbon black, and nickel powder in a mass ratio of 7:1:10. The commercial porous zinc anode mentioned in this paper was obtained from disassembled commercial zinc-nickel battery too. A nickel-plated copper mesh was employed as current collector inside the electrode. The anodes were immersed in deionized water for 1 h to remove the residual electrolyte from original battery, and then dried up before being placed into the experimental mold. The commercial zinc anode also underwent the formation process before cycle, and the conditions were the same as the experimental group.

Experimental mold and slurry renewal. A simple mold was designed to detect the performance of SSE by matching ZSSE with commercial NiOOH positive electrode. The positive electrode size was 30 mm×30 mm, while the negative electrode size as 15 mm×15 mm. To reflect the negative electrode performance, the designed capacity of the positive electrode was much higher than that of negative electrode. Tin plate was used for the current collector of negative electrode. The excess bare part was sealed by BOPP tape to prevent the electrodeposition of metal zinc on the other part of current collector

causing additional capacity. The mold was divided into a positive electrode chamber and a negative electrode chamber by a porous PP separator. The thickness of the negative electrode chamber is designed to be 0.46 mm, ensuring that the height of the sedimentary slurry was exactly 15 mm. The volume of sedimentary slurry in chamber was ~103.5 mm^3, and the total volume of the model chamber is ~ 3645 mm^3 including an anode, a cathode and excess electrolyte. The function of glass fiber membrane which wrapped the positive electrode was to absorb the electrolyte. The renewal of slurry was conducted per certain cycles (20 or 5 cycels) as mentioned before.

Characterization. The morphologies were captured by scanning electron microscopy (SEM, JEOL, JSM-5612LV). Electrochemical impendence spectroscopy (EIS) tests were investigated by CHI660d electrochemical work station.
